# Transplantation of bioengineered Reelin‐loaded PLGA/PEG micelles can accelerate neural tissue regeneration in photothrombotic stroke model of mouse

**DOI:** 10.1002/btm2.10264

**Published:** 2021-10-29

**Authors:** Zahra Shabani, Reza Rahbarghazi, Mohammad Karimipour, Tahereh Ghadiri, Roya Salehi, Saeed Sadigh‐Eteghad, Mehdi Farhoudi

**Affiliations:** ^1^ Neurosciences Research Center (NSRC) Tabriz University of Medical Sciences Tabriz Iran; ^2^ Department of Neurosciences, Faculty of Advanced Medical Sciences Tabriz University of Medical Sciences Tabriz Iran; ^3^ Stem Cell Research Center Tabriz University of Medical Sciences Tabriz Iran; ^4^ Department of Applied Cell Sciences, Faculty of Advanced Medical Sciences Tabriz University of Medical Sciences Tabriz Iran; ^5^ Department of Anatomical Sciences, Faculty of Medicine Tabriz University of Medical Sciences Tabriz Iran; ^6^ Department of Medical Nanotechnology, Faculty of Advanced Medical Sciences Tabriz University of Medical Sciences Tabriz Iran

**Keywords:** functional regeneration, neural stem cells, photothrombotic stroke, PLGA‐PEG, Reelin

## Abstract

Ischemic stroke is characterized by extensive neuronal loss, glial scar formation, neural tissue degeneration that leading to profound changes in the extracellular matrix, neuronal circuitry, and long‐lasting functional disabilities. Although transplanted neural stem cells (NSCs) can recover some of the functional deficit after stroke, retrieval is not complete and repair of lost tissue is negligible. Therefore, the current challenge is to use the combination of NSCs with suitably enriched biomaterials to retain these cells within the infarct cavity and accelerate the formation of a de novo tissue. This study aimed to test the regenerative potential of polylactic‐co‐glycolic acid‐polyethylene glycol (PLGA‐PEG) micelle biomaterial enriched with Reelin and embryonic NSCs on photothrombotic stroke model of mice to gain appropriate methods in tissue engineering. For this purpose, two sets of experiments, either in vitro or in vivo models, were performed. In vitro analyses exhibited PLGA‐PEG plus Reelin‐induced proliferation rate (Ki‐67^+^ NSCs) and neurite outgrowth (axonization and dendritization) compared to PLGA‐PEG + NSCs and Reelin + NSCs groups (*p* < 0.05). Besides, neural differentiation (Map‐2^+^ cells) was high in NSCs cultured in the presence of Reelin‐loaded PLGA‐PEG micelles (*p* < 0.05). Double immunofluorescence staining showed that Reelin‐loaded PLGA‐PEG micelles increased the number of migrating neural progenitor cells (DCX^+^ cells) and mature neurons (NeuN^+^ cells) around the lesion site compared to the groups received PLGA‐PEG and Reelin alone after 1 month (*p* < 0.05). Immunohistochemistry results showed that the PLGA/PEG plus Reelin significantly decreased the astrocytic gliosis and increased local angiogenesis (vWF‐positive cells) relative to the other groups. These changes led to the reduction of cavity size in the Reelin‐loaded PLGA‐PEG+NSCs group. Neurobehavioral tests indicated Reelin‐loaded PLGA‐PEG+NSCs promoted neurological outcome and functional recovery (*p* < 0.05). These results indicated that Reelin‐loaded PLGA‐PEG is capable of promoting NSCs dynamic growth, neuronal differentiation, and local angiogenesis following ischemic injury via providing a desirable microenvironment. These features can lead to neural tissue regeneration and functional recovery.

## 1 INTRODUCTION

Despite numerous pharmacological and neuro‐rehabilitation approaches used to recuperate the neurological disorders, stroke is still the leading neurological disability with a high mortality rate.^1^ Statistics have shown near 3.4 million people will suffer a stroke by 2030 in the United States.^2^ The lack of efficient medical treatment to restore the function of injured areas in the brain after the stroke imposes considerable economic and health system problems; hence, emerges an urgent need for a novel therapeutic approaches.^3^ Regeneration of injured brain tissue via transplanting neural stem cells (NSCs) seems to be effective in patients with ischemic disease.^4^ NSCs possess auto self‐renewability and target orientation toward main neuroectodermal lineages like oligodendrocytes, neurons, and astrocytes. In the embryonic stage, the existence of NSCs exists in mammalian neural tube which is known as ancestors in the central nervous system (CNS).^5^ By contrast, these cells are restricted to specific regions in adults located in the subventricular zone (SVZ) and the subgranular zone of the dentate gyrus (DG). It was suggested that NSCs can be distinguished from other cell lineages based on specific markers such as Nestin and glial fibrillary acidic protein (GFAP).^6^, ^7^

The promotion of magnificent neuron death in the ischemic area and consequent pathological remodeling lead to the formation of large blank spaces, which necessitate the application of bulk volume transplants and grafts using stem cells.^3^ This area is juxtaposed to peri‐infarct tissue known as penumbra where both neural plasticity and angiogenesis are prominent.^8^

Although many in vitro protocols are effective in the orientation of NSCs toward targeted lineages, the control of NSCs phenotype acquisition is not possible in in vivo conditions.^3^ On this basis, several cell delivery approaches have been limited because of the low viability of transplanted stem cells,^9^ and the transient activity of growth factors.^10^, ^11^ The initiation of inflammatory response and lack of adhesive support, to some extent, have forced researchers to find alternative therapeutic approaches.^12^, ^13^ Accordingly, considering the critical role of distinct factors can help the scientists in an efficient NSC‐based modality. It is noteworthy to mention that NSCs alone are not potent enough to generate nascent functional neurons and undergo atretic changes soon after transplantation due to the lack of supporting extracellular matrix (ECM). It has been shown that NSCs cannot migrate to the depth of the stroke site due to a lack of signaling ECM. By providing sufficient structural support and substrates, it is applicable to increase NSCs recruitment to the stroke‐damaged area.^14^, ^15^ To this end, recent findings have developed scaffolds and hydrogels for the transplantation of NSCs with the capability to promote simultaneously the interaction between transplanted NSCs and surrounding matrix.^16^

Among different ECM components, Reelin can regulate the migration of neurons during the developmental period of the brain. This protein is touted as a key player in the formation of the cerebral cortex and lamination of the cerebellum,^17^ and maintenance of adult synaptogenesis.^18^ Likewise, it was suggested that Reelin can reduce pathologies related to cerebral ischemia–reperfusion injury. In support of this claim, the suppression of Reelin increases vulnerability after cerebral ischemia in mouse models.^19^ Moreover, Reelin organizes vascular morphology and orientation and induces vascular sprouting and vascular wall integrity.^20^, ^21^ Therefore, the induction of Reelin synthesis in acute CNS injuries can help us to minimize clinically the progression of pathologies after ischemic changes.^22^ Although natural ECM components can regulate cell bioactivity the combination of these factors with synthesized scaffolds yielded an appropriate regenerative outcome via supporting structural properties.^23^

One key artificial FDA‐approved biomaterial is polylactic‐co‐glycolic acid (PLGA) that can be injected directly into the injured sites. Due to certain and unique physicochemical features, PLGA can structurally support stem cells during delivery.^24^ Given the role of PLGA nanoparticles as a potentially promising carrier for the treatment of brain disorders, unmodified PLGA possesses numerous weaknesses, such as negative charge, hydrophobic structure, and the existence of free glycolic subunits. Accordingly, the PLGA nanoparticle fails to interact appropriately with the diverse cells due to hydrophobicity.^25^, ^26^, ^27^ Along with these comments, self‐assembled micellar nanoparticles with hydrophilic shells and hydrophobic cores can increase the solubility of hydrophobic biomolecules. Besides, the existence of nanoparticle corona enclosed by hydrophilic units maintains the structure of target molecules in different microenvironments.^28^, ^29^ Hence, surface modification and additional engineering of PLGA nanoparticles are highly demanded. One important avenue for PLGA functionalization is the attachment of polyethylene glycol (PEG) polymer chains known also as PEGylation.^25^ Therefore, the combining PLGA copolymer with non‐cytotoxic and hydrophilic PEG substrate offers the desired microenvironment for the generation of perineuronal net.^30^

Here, we fabricated PLGA‐PEG micelles loaded with Reelin for triggering neurogenesis either in in vivo and in vitro conditions. Besides, we explored the regeneration of ischemic brain injury and functional recovery in the photothrombotic stroke model of the mouse.

## 2 MATERIALS AND METHODS

### 2.1 Preparation of PLGA‐PEG polymer

PEG (1 g) with a molecular weight of 4000 was kept at 150°C for 3 ho under a high vacuum nitrogen atmosphere. Concurrently, DL‐lactide (0.85 g) and glycolide (0.15 g) with a mole ratio of 85:15 were mixed with PEG and melted under argon flow. Following the addition of stannous 2‐ethyl hexanoate, the mixture was maintained at 155°C for 8 h to initiate the reaction. This reaction was performed under a relative vacuum. After the completion of the reaction, we declined the temperature and adjusted to room temperature. The synthesized polymer was dissolved in dichloromethane solution followed by the precipitation in pre‐cold diethyl ether solution. The procedure was followed by polymer dehydration at a vacuum oven at RT.

### 2.2 Synthesis and development of PLGA‐PEG micelles

Blank polymeric micelles were developed by dissolving 2 mg polymer in dimethyl sulfoxide (60 μl, dimethyl sulfoxide [DMSO]). Then, this solution was carefully dropwise to 200 μl PVA (polyvinyl alcohol) 1% w/v with sonication. Synthetized micelles were collected by centrifugal filters (molecular weight cutoff of 100 kDa) at 4000 rpm for 10 min and freeze‐dried. To synthesize Reelin‐loaded micelles, 2 mg polymer solution dissolved in 60 μl DMSO was added drop‐wise to 200 μl PVA (1%) solution containing 25 μg Reelin (Cat no: 3820‐MR‐025, R&D Systems). Then, the pH of the PVA–Reelin solution pH was set to 7.4. Eventually, the Reelin‐loaded micelles were collected using centrifugal filter tubes as above mentioned.

### 2.3 Materials characterization

#### 2.3.1 Hydrogen‐1 nuclear magnetic resonance

Hydrogen‐1 nuclear magnetic resonance (^1^H‐NMR) analysis was applied to analyze the composition of micelles. For this purpose, the obtained spectra were collected from CDCI3 Brucker AM 300.13 MHz spectrometers (Germany).

#### 2.3.2 Fourier transforms infrared

The composition of PLGA‐PEG and Reelin‐loaded PLGA‐PEG micelles was analyzed by Fourier transforms infrared (FTIR) spectroscopy (Equinox 55 LS 101, Germany). The samples were scanned in the range of 400 up to 4000 cm^−1^.

#### 2.3.3 Dynamic light scattering and scanning electron microscope

Particle size and net charge of PLGA‐PEG micelles were evaluated by dynamic light scattering (DLS) (Malvern, UK). For the DLS analysis, PLGA‐PEG micelles were dissolved in deionized water with the final concentration of 5 mg/ml at room temperature. Using scanning electron microscope (SEM) (Model: Tescan Mira3, Czech), we measured the morphology and diameter of fabricated micelles. After gold sputtering, the diameter of micelles was obtained by monitoring 100 particles and raw data processed using Image‐Pro plus 4.5 (Silver Spring, MD).

### 2.4 Bradford assay

The releasing content of Reelin‐loaded PLGA‐PEG micelles was assessed using Bradford assay. To this end, the micelles were incubated for 7 days. A 25 μg Reelin was incubated with 2500 μg PLGA‐PEG micelles in 1 ml of fetal bovine serum (FBS)‐free culture medium for 7 days. After the completion of incubation time, the micelles were collected and protein content was assessed using Bradford solution. Data were read at a wavelength of 595 nm using a microplate reader and compared to the PLGA‐PEG micelles without Reelin treatment.

### 2.5 Animal housing and ethics

Adult male mice (8–12 weeks) weighing (20–22 g) were maintained in the Neuroscience Research Center affiliated to Tabriz University of Medical Sciences for 14 days before the initiation of experimental procedures. Prior and post‐surgery procedures, mice were maintained separately in standard cages. Animals were kept on a 12 h light/dark cycle 23 ± 1°C with free access to food and water. This study was performed following principal guidelines published and approved by a Local Ethics Committee of Tabriz University of Medical Sciences (approval number: 60232).

### 2.6 Experimental design

In this study, we performed both in vitro and in vivo experiments to assess neurogenesis, regenerative potential, and functional efficiency of Reelin‐loaded PLGA‐PEG micelles.

#### 2.6.1 In vitro assays

##### NSCs isolation and expansion

To this end, we isolated NSCs from nine 14‐day‐old murine embryos in three technical replicates. Pregnant mice were euthanized by cervical dislocation. Next, embryos ganglionic eminences were dissected using fine scissors and forceps under the operating microscope. Ganglion eminences were incubated in sterile Dulbecco's Modified Eagle Medium/Nutrient Mixture (DMEM/F‐12; Cat no: 21331‐020; Gibco) supplemented with 5% Pen‐Strep (Cat no: 15140122; Gibco), chopped carefully and enzymatically digested by 0.05% Trypsin–EDTA (Gibco) for 3 min to yield single‐cell suspension.^31^, ^32^ Thereafter, samples were centrifuged at 700 rpm for 5 min and collected cells were cultured in DMEM/F‐12. The medium was enriched with 1% Pen‐Strep, 2% B27 (Cat no: 17504044; Gibco), 20 ng/ml epidermal growth factor (EGF) (Gibco), 10 ng/ml basic fibroblast growth factor‐2 (FGF‐2, Gibco), and 2 μg/ml heparin. Freshly isolated cells were cultured (50,000 cells/cm^2^) at 37°C with 5% CO_2_ inside the humidified incubator for 7 to 10 days. During the period, NSCs generated neurospheres with a diameter reaching 200–250 μm.^33^, ^34^ After the first passage, neurospheres were passaged every 5–7 days. At this time, NSCs at passages 3–5 were subjected to in vitro and in vivo analyses.^32^

##### Flow cytometric analysis of Nestin in NSCs

The stemness feature of NSCs was assessed in expanded NSCs by monitoring Nestin levels. For this purpose, NSCs at passage 3 with 80%–90% confluence were detached using the enzymatic solution and submitted to the flow cytometry analysis. 1 × 10^6^ cells were blocked with 1% BSA and incubated with 1 μg/mL FITC‐conjugated Nestin antibody (Biosciences) at 4°C for 45 min. Finally, the cells were analyzed using a FACSCalibur cytometer and FlowJo software version 7.6.1.^35^

##### MTT assay

To this end, the possible cytotoxic effect of PLGA‐PEG micelles and Reelin was investigated on NSCs using MTT assay. NSCs were incubated with different concentrations of PLGA‐PEG micelles including 50, 100, 250, and 500 ng/ml, and survival rate determined after 1, 2, 3, 7, 14, and 21 days. In another setting, NSCs were incubated with different doses of Reelin including 50, 100, and 200 μM for similar time points. About 1 × 10^4^ NSCs were re‐suspended in 100 μL DMEM/F12 and transferred into the 96‐well plates and kept at standard conditions. After completion of experimental periods, the supernatants were discarded and replaced with 20 μl MTT (5 mg/ml dissolved in PBS). Plates were maintained at 37°C for 3–4 h. The procedure was continued with the addition of 100 μl DMSO. Finally, the ODs were read at 570 nm using a microplate reader. The viability was expressed as % of control.^36^, ^37^

##### Measuring proliferation and differentiation capacity of NSCs

We investigated the proliferation rate and differentiation capacity of NSCs after being cultured on Reelin‐loaded PLGA‐PEG micelles using immunofluorescence (IF) assays. In brief, NSCs were allocated into Control; PLGA/PEG; Reelin; and PLGA/PEG + Reelin groups. In order to evaluate proliferation rate and differentiation capacity, NSCs were cultured at a density of 50,000/ml per well of 24‐well plates pre‐coated with laminin. On day 7, the proliferation rate was monitored in terms of nuclear Ki‐67 protein levels. In line with this assay, we studied differentiation capacity of cultured NSCs on different substrates by monitoring Map‐2 levels after 14 days. The proliferation medium consists of basic medium‐plus bFGF, and EGF while the differentiation medium was supplemented with 5% FBS in the absence of bFGF, and EGF. After completion of experimental periods, cells were fixed by 4% (w/v) pre‐cold paraformaldehyde (PFA) for 20 min and washed with PBS. To reduce non‐specific binding, NSCs were blocked with 1% BSA for 20 min followed by permeabilization with 0.1% Triton X100. For proliferation and differentiation assays, cells were incubated overnight with rabbit anti‐Ki67 (1:500, Abcam) and anti‐MAP‐2 (1:500; Sigma‐Aldrich) antibodies, respectively. After PBS washes, cells were incubated with Alexa Fluor® 488‐conjugated secondary antibody for 1 h at room temperature. A 1 μg/mL DAPI was used to stain nuclei. In this study, the number of Ki‐67^+^ and MAP‐2^+^ cells was counted per 100 DAPI^+^ cells.^38^, ^39^

##### Neurite formation analysis

Neurite formation was also studied in NSCs cultured on three different surfaces including PLGA/PEG; Reeling and PLGA/PEG + Reeling. Parameters such as the number of primary neurites, branch points, and length of neurite per cell were measured using SEM and bright‐field imaging. The cells cultured on plastic surfaces were considered as a control group. The length of neurites was calculated by using ImageJ software version 1.4 (NIH).

### 2.7 In vivo setting

#### 2.7.1 Induction of photothrombotic stroke model

For in vivo analyses, a total 50 male Balb/C mice were assigned randomly into five groups (each in 12) as follows; stroke + PBS; stroke + NSCs; stroke + PLGA/PEG + NSCs; stroke + Reelin + NSCs; Stroke + PLGA/PEG + Reelin + NSCs. Mice were anesthetized using 5% isoflurane gas with a rate of 1.5 L/min and carefully placed onto a stereotactic apparatus (Stoelting, USA). Following shaving skull hair, a longitudinal incision (1.0–1.5 cm) was generated from surface to depth using a surgical blade to expose coronal and sagittal sutures. The interest site with the feature of [AP]: 1.1 mm, [ML]: 2 mm was targeted using the stereotaxic atlas of Paxinos and Watson. Before photothrombotic stroke induction, 150 μg sterile Rose Bengal/g, dissolved in normal saline, was injected per g/body weight and allowed to distribute into the blood circulation. In this study, the light green laser was irradiated on the skull surface for 10 min followed by wound suturing. Thereafter, mice were maintained at temperature‐controlled conditions. To examine the efficiency of our protocol in the induction of ischemic stroke, we selected mice randomly from different groups immediately after laser irradiation. Mice were euthanized by an overdose of ketamine and xylazine. The brains were removed, coronal sections prepared with identical intervals, and stained with 2, 3, 5‐triphenyl‐tetrazolium chloride (TTC) solution. We monitored general appearance and morphological features.

#### 2.7.2 Cell labeling

To track transplanted NSCs after injection into the brain tissue, we labeled cells using Cell Tracker™ CM‐DiI Dye. To this end, the supernatant medium was removed and NSCs were incubated with 20 μM dye solution for 20 min at 37°C. After three PBS washes, cells were ready for injection.

#### 2.7.3 Cell transplantation

Seven days after the laser irradiation, mice from different groups were again undergone deep anesthesia and were placed onto a stereotactic apparatus. A small hole of diameter 2 mm was induced at the same location that was previously irradiated. The procedure was continued by the insertion of a cannula at a depth of 1.3 mm from the dural surface. In all groups, the volume injection was 5 μl in bregma coordinates ([AP]: 1.1 mm, [ML]: 2 mm) using a 24‐gauge needle. The injection rate was adjusted to 1 μl per minute. Upon completion of the injection process, the needle was left at the injection site near 5 min to prevent suspension backflow. NSCs were transplanted at a concentration of 5 × 10^4^ cells/μl. In groups that received hydrogel, 20 μg/μl PLGA‐PEG micelles we used. In this study, the final concentration of Reelin reached 200 nM.

#### 2.7.4 Unbiased stereological estimation

To perform stereological evaluation, three mice per group were chosen on 7 and 28 days after stroke induction and transplantation, respectively. Animals were euthanized using the overdose of ketamine and xylazine and perfused using normal saline and 4% PFA in 0.01 M phosphate buffer. The samples were again incubated in 4% PFA overnight at 4°C. The procedure was continued with dehydration of samples in serial increasing alcohol solutions for clearing, and paraffin embedding. Twelve serial coronal sections were performed and selected using a systematic uniform random sampling design and stained with 0.1% Cresyl violet solution. The cavity size was measured according to Cavalier's principle using the point‐counting method.^40^, ^41^, ^42^

#### 2.7.5 Double Dil/DCX and Dil/NeuN IF staining

For IF analysis, prepared brain samples 14 and 28 days after transplantation were selected. Following paraffin embedding, sections with thicknesses of 5 μm were prepared and antigen retrieval was performed by incubating sections in pre‐heated 10 mM sodium citrate buffer at 100°C for 15 min. To avoid unspecific binding, sections were incubated in a blocking solution containing 0.1% Triton X‐100 and 5% BSA for 30 min at room temperature. Then samples were incubated with anti‐mouse doublecortin (DCX) (1:50) and anti‐mouse NeuN (1:100) at 4°C overnight. After PBS washes, goat anti‐mouse conjugated with Alex Flour 488 for 2 h at room temperature. DAPI was used to stain nuclei and percent of DCX^+^ and NeuN^+^/Dil^+^ cells were calculated and visualized under fluorescence microscopy.

#### 2.7.6 Immunohistochemistry analysis used for evaluation of astrocytic gliosis and local angiogenesis

On day 28, the prepared slides (as above‐mentioned) were used for Immunohistochemistry (IHC) analysis of GFAP positive astrocytes. In brief, the endogenous activity was inhibited by using 3% H_2_O_2_ for 20 min after antigen retrieval. The slides were exposed to anti‐GFAP and‐von Willebrand factor (vWF) antibodies (Dako) for 1 h at room temperature and washed three times with PBS. The procedure was continued with the addition of the EnVision + Dual Link System HRP kit (Dako). DAB was exploited as chromagen. The number of GFAP‐positive astrocytes and vWF‐positive endothelial cells were calculated in the periphery of the injured area in different groups and compared to the control stroke mice. To this end, we took serial images with high magnification from the periphery of the injured area of each slide. Then, the periphery zones were randomly outlined into five subfields and the number of vWF‐ and GFAP‐positive cells were counted and compared to the control group.

#### 2.7.7 Behavioral performance assessment

##### Modified neurological severity score (mNSS)

One day after stroke induction and 7, 14, 21, and 28 days after transplantation, the mNSS test was performed to assess behavioral performance. All values correlated with motor and sensory functions such as general movements, balance, and reflex were measured on a scale system consisted of minimal normal (0) and maximal deficit (18) scores. Score 0 stands for a fact that neurological deficit does not exist, whereas score 18 shows the maximum injury of CNS following stroke. Scores between these values were classified as follows; 0–6: mild neurological deficit; 7–12: moderate neurological impairment, and 13–18: the severe neurological deficit. The functionality of the motor system was evaluated by suspending the mice from the tail to monitor head movements and forelimbs flexion in the vertical axis. Mice were placed on a flat surface for assessing gait.^43^, ^44^ In addition to motor system function, the function of the sensory system was studied by evaluating both visual, tactile, and proprioception senses.^43^, ^45^, ^46^ According to previously published data, the status of balance was assessed as a slim wooden with 100 cm away from the ground. Different reflexes like pinna, corneal, and startle reflexes were scored. We also investigated myodystonia, myoclonus, and seizure in mice from all groups.

### 2.8 Statistical analysis

Data were represented as mean ± SD. The statistical differences between groups were analyzed using One‐way ANOVA with post hoc Tukey (GraphPad Prism Version 8) otherwise mentioned. *p* < 0.05 was considered statistically significant.

## 3 RESULTS

### 3.1 H‐NMR spectrum

**FIGURE 1:**
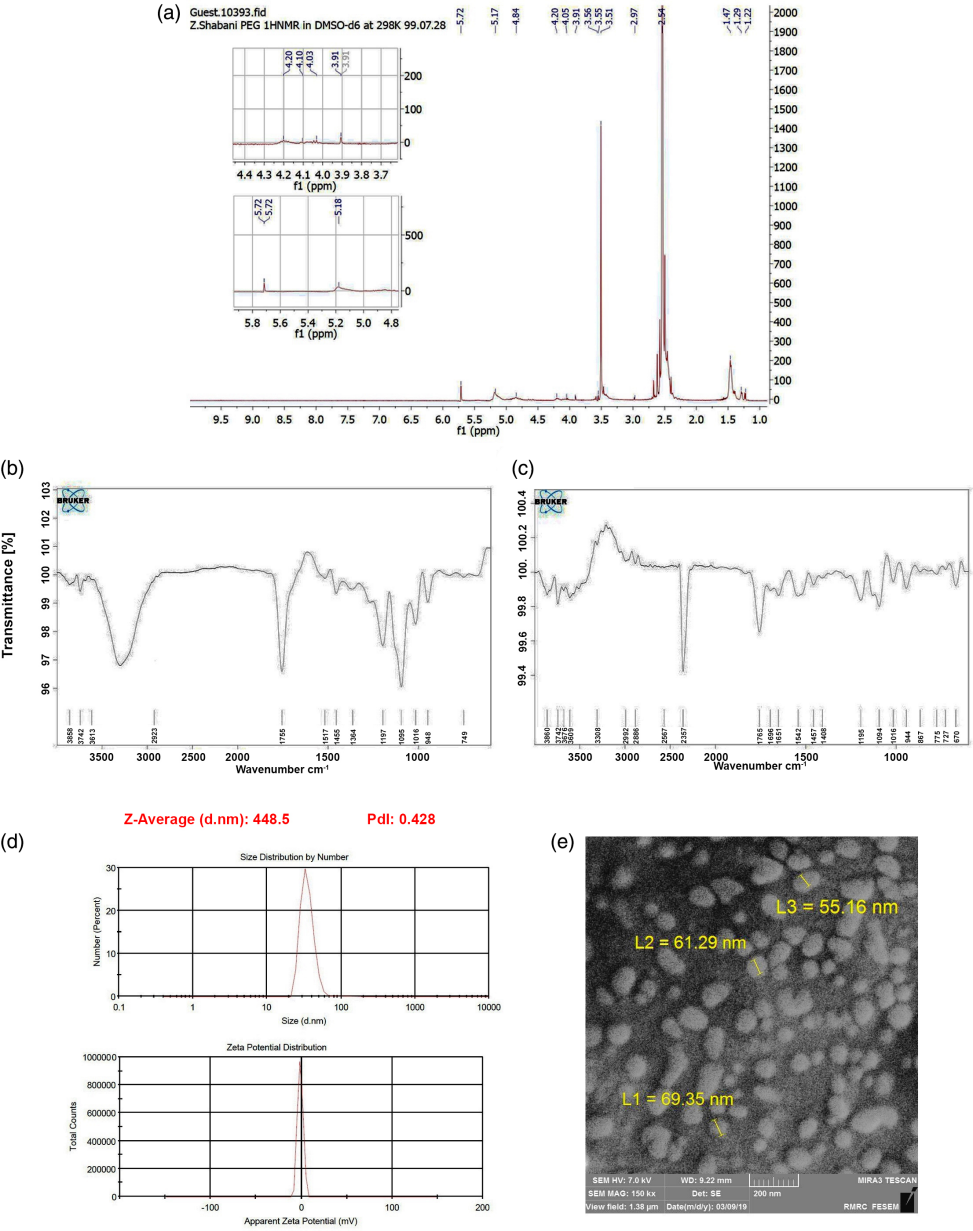
Chemical characterization of synthesized biomaterials. ^H^NMR spectra revealed successful conjugation of PEG to the PLGA backbone (a). FTIR spectroscopy analysis of PLGA‐PEG (b) and Reelin‐loaded PLGA‐PEG micelles (c). Data showed the existence of certain functional groups that are related to Reelin and PLGA‐PEG. DLS analysis exhibited that the size and zeta potential of PLGA‐PEG micelles reached 79.7 ± 45.4 nm and approximately −1.21 mV, respectively (d). SEM imaging displayed that PLGA‐PEG micelles showed a spherical‐like appearance (e). DLS, dynamic light scatteringncs; FTIR, Fourier transforms infrared; PLGA‐PEG, polylactic‐co‐glycolic acid‐polyethylene glycol

H‐NMR spectra exhibited peaks at 5.3 and 4.8 ppm correlated with CH and CH_2_ groups for lactide and the glycolide, respectively (Figure 1a). According to our data, the peak at 1.47 ppm is associated with lactide CH_3_ while the peak of 3.5 ppm confirms the existence of methylene groups related to PEG (Figure 1a). The data demonstrate successful synthesis of PEG and PEG‐PLGA conjugation.

### 3.2 FT‐IR analysis

FT‐IR spectra of the PLGA‐PEG (Figure 1b) and Reelin‐loaded PLGA‐PEG (Figure 1c) micelles were performed in this study. We noted peaks at 1755, 1197, and 1095 cm^−1^ which are associated with C═O and C—C—O, and etheric C—O—C bonds. Carboxylic acid groups were observable at 3300–3400 cm^−1^. The existence of a bond at 2923 cm^−1^ is associated with the C—H group of ethylene glycol (Figure 1b). In Reelin‐loaded PLGA‐PEG, amide I and carboxylic acid groups were determined at 1651 and 1696 cm^−1^ (Figure 1c).

### 3.3 DLS analysis and morphological assessment by SEM

DLS analysis was used to measure zeta potential and micelle size (Figure 1d). Data showed a mean size of 79.7 ± 45.4 nm and approximately −1.21 mV zeta potential for PLGA‐PEG micelles. SEM analysis displayed relatively homogenous spherical morphology in PLGA‐PEG micelles (Figure 1d). Based on our analysis, the mean diameter of micelles was reached 80.28 ± 22.72 nm. We noted polydispersity index of 0.428 for developed micelles. The existence of a semi‐spherical appearance showed the efficiency of our protocol in the fabrication of PLGA‐PEG micelles.

### 3.4 Reelin‐loaded PLGA‐PEG micelles can release Reelin in aqueous phase

**FIGURE 2:**
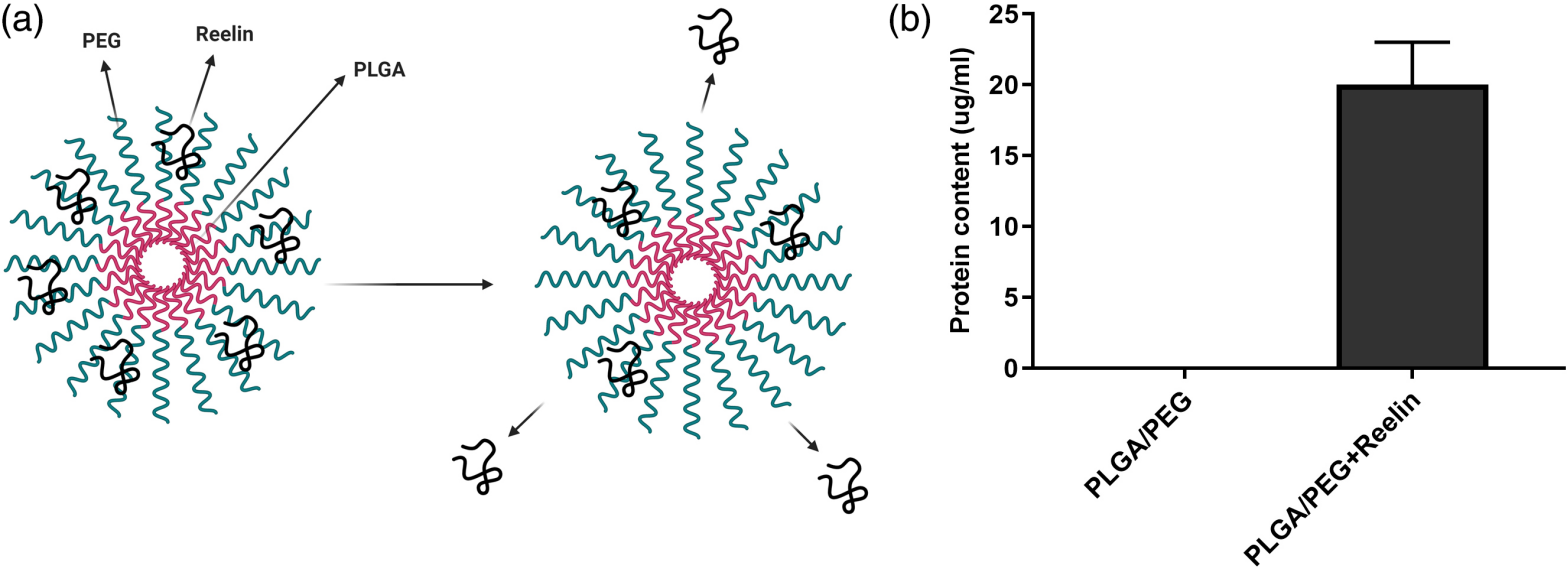
Measuring the content of Reelin after loading onto PLGA‐PEG micelles (a,b). Schematic representation of Reelin‐loaded PLGA‐PEG and release of Reelin from the substrate (a). Bradford assay indicated that PLGA‐PEG micelles can adsorb Reelin peptide after 7‐day incubation compared to the Reelin‐free PLGA‐PEG micelles. PLGA‐PEG, polylactic‐co‐glycolic acid‐polyethylene glycol

Here, we measured the releasing capacity of Reelin after blending with PLGA‐PEG micelles using the Bradford assay (Figure 2a,b). We blended 25 μg Reelin peptide with 2500 μg PLGA‐PEG micelles and releasing capacity was measured after seven‐day incubation inside culture medium. Here, we found that the released content of total protein reached 20 ± 3 μg compared to the Reelin‐free PLGA‐PEG micelles. This protocol yielded an 80% loading rate which seems suitable for Reelin peptide delivery to the target cells and tissues.

### 3.5 NSCs morphology and characterization

**FIGURE 3:**
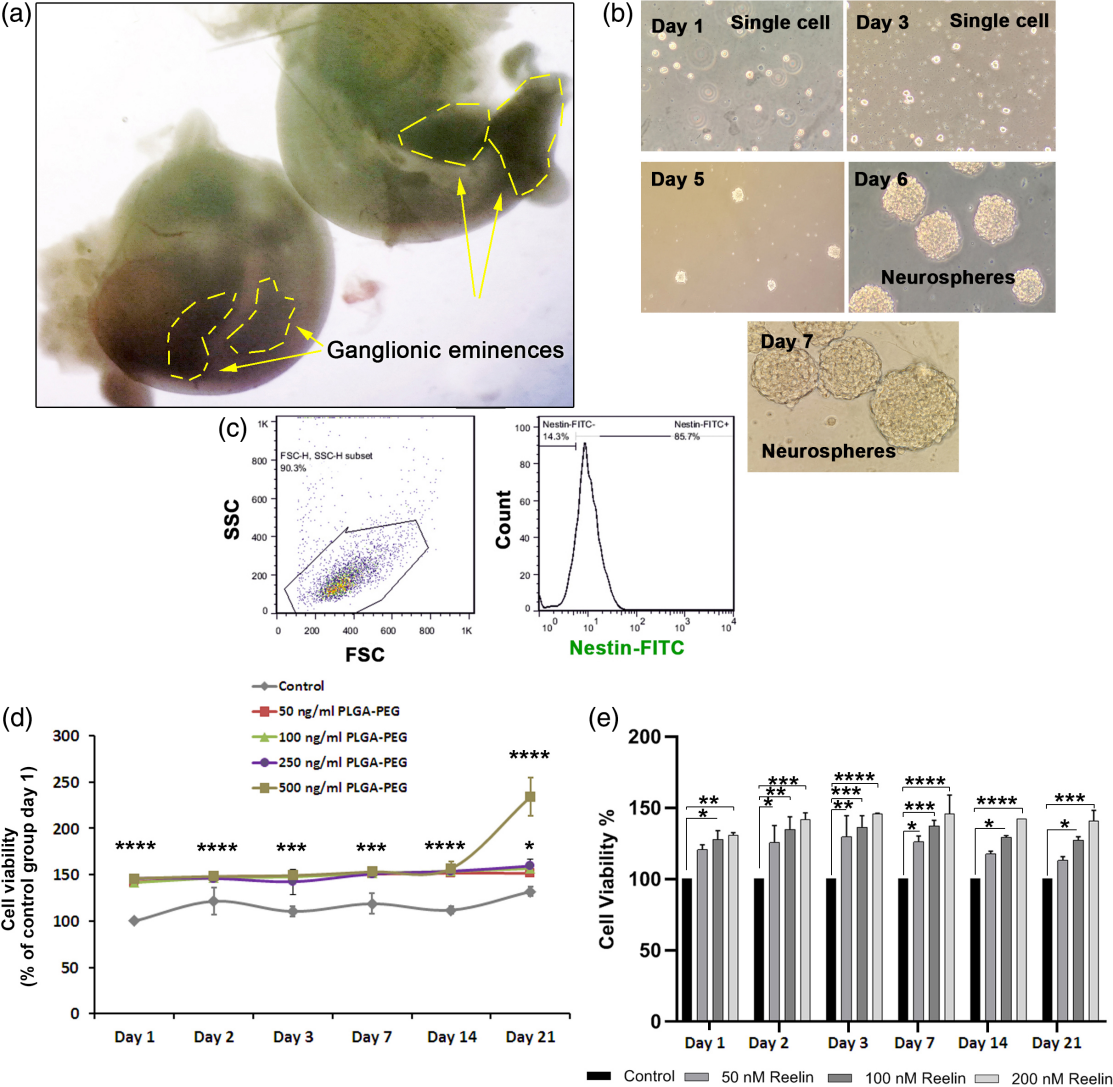
Isolation of mouse embryonic NSCs from ganglionic eminence (a). Bright‐field imaging revealed the formation of neurospheres during the first 7 days. Data showed that isolated cells can form distinct neurospheres 6 days after plating in vitro (b). Flow cytometry analysis confirmed that about 85% of cultured NSCs were positive for nestin, indicating the efficiency of our protocol in the isolation of mouse NSCs (c). MTT assay (d). NSC viability was significantly increased after exposure to 100, 250, and 500 ng/mL PLGA‐PEG micelles for 21 days compared to the nontreated control NSCs. MTT assay showed that Reelin can increase NSC survival rate in a dose‐dependent manner after 21 days. One‐way ANOVA and Tukey post hoc analysis. *p < 0.05; **p < 0.01; ***p < 0.001; and ****p < 0.0001. NSC, neural stem cells; PLGA‐PEG, polylactic‐co‐glycolic acid‐polyethylene glycol

In this study, we isolated NSCs from ganglionic eminences of E14 brains (Figure 2a). Bright‐field imaging revealed that small‐sized colonies were generated on Day 5 after plating. These structures were enlarged to perform mature neurospheres in which Days 6–10 their diameters reached 200–250 μm (Figure 2b). After three consecutive passages, NSCs were subjected to flow cytometry analysis (Figure 3c). According to our data, over 85% of cells were Nestin positive, indicating the efficiency of our protocol in NSCs isolation and enrichment.

### 3.6 PLGA‐PEG micelles and Reelin increased NSCs viability

To evaluate the possible cytotoxicity of PLGA‐PEG micelles on mouse NSCs, we performed an MTT assay (Figure 3d). To this end, NSCs survival rate was determined on days 1, 2, 3, 7, 14, and 21 exposed to different concentrations of PLGA‐PEG micelles including 50, 100, 250, and 500 ng/ml. We found NSCs treated with all concentrations of PLGA‐PEG micelle displayed enhanced survival rates in all‐time points compared to the control group (*p* < 0.05; Figure 3d). No statistically significant differences were found between groups incubated with several doses of PLGA‐PEG micelles in all‐time points except 500 ng/ml PLGA‐PEG micelle group on day 21 (*p* < 0.05). Based on the obtained data, the 500 ng/ml PLGA‐PEG micelle group significantly increased NSCs viability compared to the control NSCs and other groups (*p* < 0.05). These data demonstrated that certain doses of PLGA‐PEG micelles had potential to statistically increase survival compared to the lower doses on specific time period. In the concentration range from 50 to 250 ng/mL, the viability of NSCs was not altered even after 21 days. The viability of NSCs was also assessed in the presence of different doses of Reelin using MTT assay and compared to non‐treated control at each time points (Figure 3e). We noted that NSCs survival rate was induced by increasing the dose of Reeling from 50 to 200 nM in all‐time points when compared to the control‐matched NSCs groups (*p* < 0.05). To be specific, the maximum cell survival rate was achieved at each time point in group that received 200 nM Reelin compared to other groups (*p* < 0.05). According to the MTT data, we selected 200 nM Reelin for different in vitro and in vivo analyses.

### 3.7 Reelin‐loaded PLGA‐PEG micelles increased proliferation and differentiation of mouse NSCs

**FIGURE 4:**
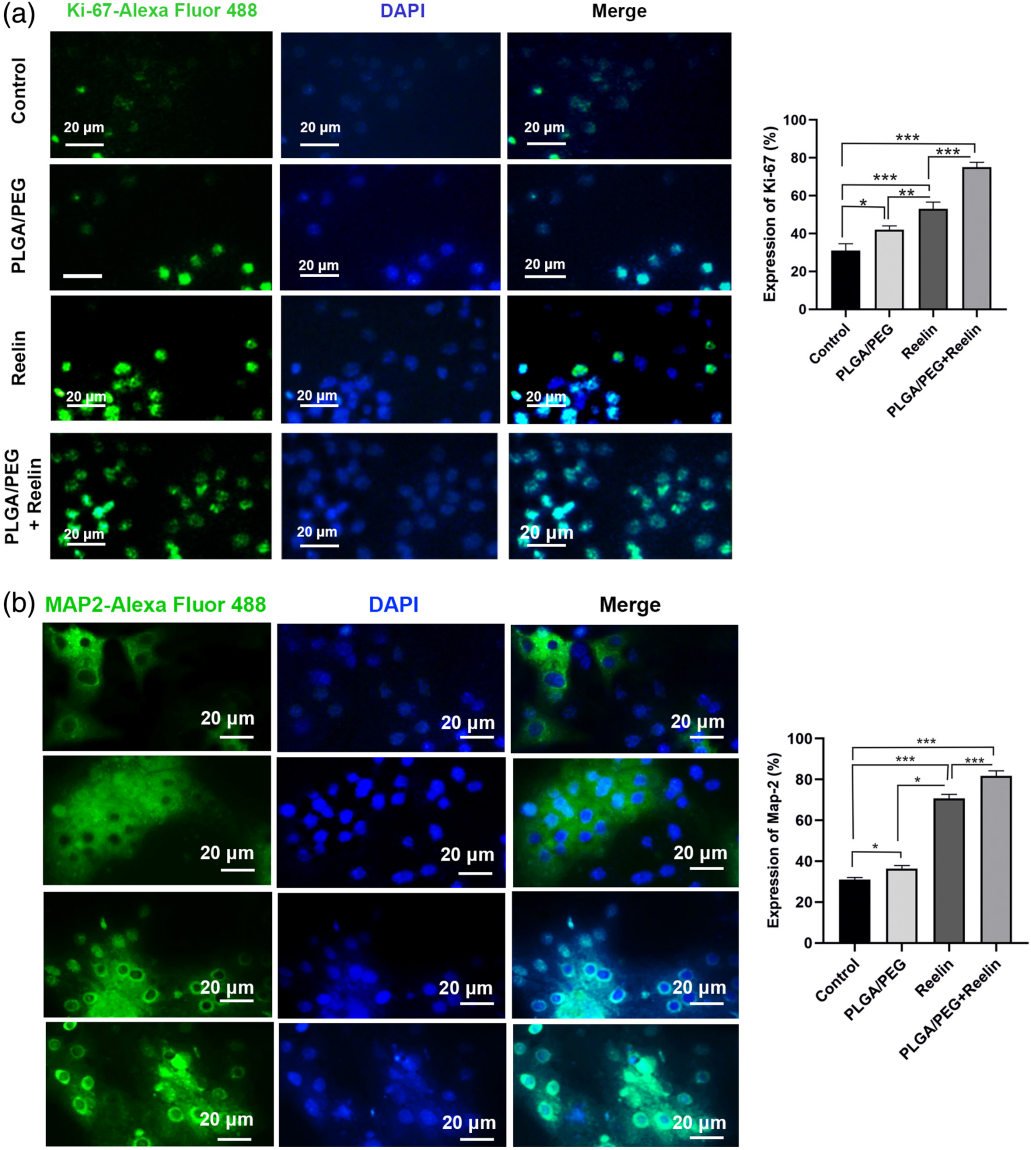
In vitro characterization of mouse NSC proliferation and differentiation using IF assay (a,b). The expression of proliferation (Ki67) and differentiation (differentiation) markers were increased in NSCs incubated with Reelin‐loaded PLGA‐PEG micelles after 7 and 14 days, respectively. These effects were less in PLGA‐PEG micelles and Reelin groups. One‐Way ANOVA and Tukey post hoc analysis. *p < 0.05; **p < 0.01; ***p < 0.001; and ****p < 0.0001. IF, immunofluorescence; NSC, neural stem cells; PLGA‐PEG, polylactic‐co‐glycolic acid‐polyethylene glycol

Both proliferation and differentiation were studied in NSCs treated with the combination of Reelin and PLGA‐PEG micelles on days 7 and 14, respectively (Figure 4a,b). Data showed that 7‐day incubation of mouse NSCs with Reelin (200 nM), PLGA‐PEG micelles, and Reelin plus PLGA‐PEG micelles increased proliferation rate (Ki‐67 positive NSCs) as compared to the non‐treated control NSCs (Figure 4a). Based on our data, we found that Reelin alone can increase the proliferation of NSCs compared to the PLGA‐PEG micelles and control groups (p < 0.05). Noteworthy, the combination of Reelin and PLGA‐PEG micelles yielded maximum effect to enter mouse NSCs proliferation in comparison with other groups (Figure 4a), showing the synergistic effect of Reelin and PLGA‐PEG micelles in dynamic growths.

We also found that the numbers of Map‐2^+^ cells were significantly increased in PLGA‐PEG, Reelin, and Reelin‐loaded PLGA‐PEG groups after 14 days compared to the NSCs (p < 0.05; Figure 4b). Compared to the PLGA‐PEG and control groups, Reelin increased the number of Map‐2^+^ cells (p < 0.05). As expected, PLGA‐PEG micelles plus Reelin led to prominent differentiation of mouse NSCs toward Map‐2^+^ neurons (Figure 4b). These data showed that the loading of Reelin on PLGA‐PEG micelle surface increased the proliferation and differentiation of mouse NSCs.

### 3.8 Reelin‐loaded PLGA‐PEG micelles improved neurite outgrowth

**FIGURE 5:**
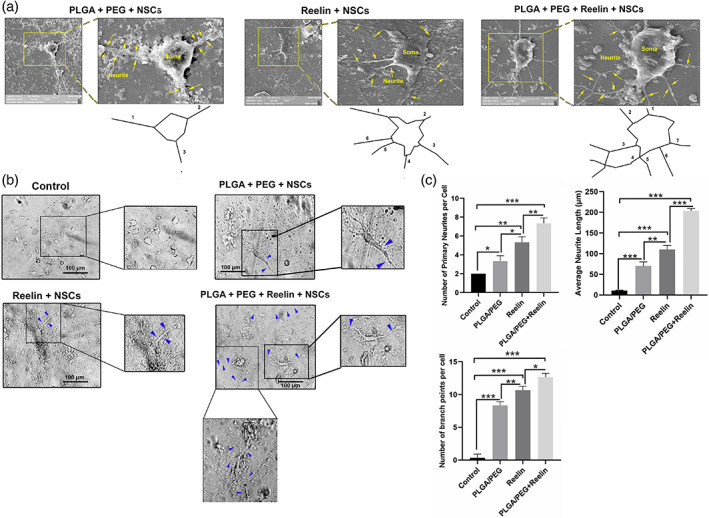
Measuring neurite growth in embryonic NSCs after 14 days culture on PLGA‐PEG micelles, Reelin, and Reelin‐loaded PLGA‐PEG micelles (a‐c). SEM imaging revealed robust neurite growth in NSCs plated on Reelin‐loaded PLGA‐PEG micelles compared to other groups (a). Consistently, bright‐field imaging showed that the mean number of primary neurites, branch points, and neurite length was significantly increased per neuron after the combination of Reelin‐loaded PLGA‐PEG micelles compared to the control NSCs (b,c). Reelin had more effects to induce neurite formation when compared to the PLGA‐PEG and control groups. Yellow arrows and blue arrowheads: primary neurites. One‐way ANOVA and Tukey post hoc analysis. *p < 0.05; **p < 0.01; and ***p < 0.001. NSC, neural stem cells; PLGA‐PEG, polylactic‐co‐glycolic acid‐polyethylene glycol

The number of primary neurites, branch points, and neurites length were analyzed per cell 14 days after plating (Figure 5a‐c). SEM analysis revealed the attachment of mouse NSCs to the PLGA‐PEG, Reelin, and Reelin‐loaded PLGA‐PEG surfaces (Figure 5a). According to our data, NSCs flattened 14 days after culture in the different substrates. Noteworthy, the extent of flattening was different in the experimental groups. Cells are round shape in the surfaces coated with PLGA‐PEG micelles whereas in groups Reelin and Reelin‐loaded PLGA‐PEG micelles NSCs are flattened (Figure 5a). In Reelin‐loaded PLGA‐PEG micelles, the number of primary neurites was increased compared to the other groups. Similarly, bright‐field imaging revealed that the number of primary neurites, branch points, and neurites length reached maximum levels in Reelin‐loaded PLGA‐PEG micelles compared to the PLGA‐PEG micelles and Reelin groups (*p* < 0.05; Figure 5b,c). These data demonstrate that the increase of cellular projection can help each cell to easily communicate with juxtaposed cells via synaptogenesis.

### 3.9 Reelin‐loaded PLGA‐PEG micelles reduced cavity size

**FIGURE 6:**
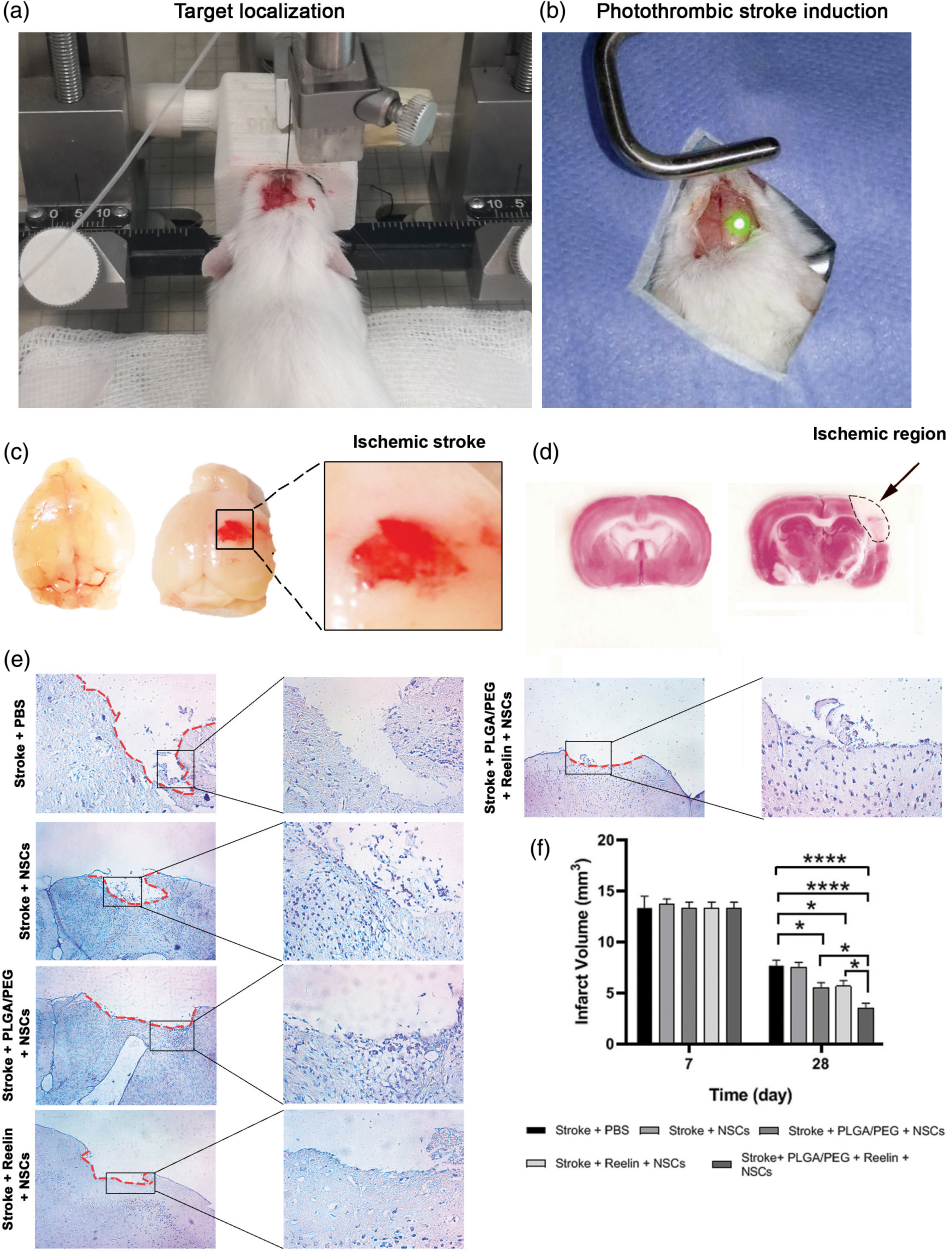
Induction of ischemic changes in mouse brain using PT (571 nm‐cold light green laser illumination) under stereotactic surgery (a,b). Macroscopic findings revealed prominent local hemorrhagia (ecchymosis) on the right hemisphere immediately after the completion of PT stroke induction (c). Longitudinal TTC staining on day 7 post‐PT stroke induction revealed a distinct ischemic area. Measuring cavity size using crystal violet staining in brain sections on days 7 and 28 after PT and after injection of PLGA‐PEG, Reelin, and Reelin‐loaded PLGA‐PEG micelles (e‐f). On Day 7, no statistically significant differences were found in lesion sites between groups while the injection of PLGA‐PEG, Reelin, and Reelin‐loaded PLGA‐PEG micelles decreased cavity size compared to the control stroke group. The injection of NSCs did not alter cavity size compared to the Stroke + PBS group. One‐Way ANOVA and Tukey post hoc analysis. *p < 0.05; and ****p < 0.0001. NSC, neural stem cells; PLGA‐PEG, polylactic‐co‐glycolic acid‐polyethylene glycol; PT, photothrombotic; TTC, 2, 3, 5‐triphenyl‐tetrazolium chloride

In this study, we used focal photothrombotic stroke model of the mouse (Figure 6a,b). Gross appearance exhibited focal ischemia immediately after laser irradiation at the right hemispheres (Figure 6C). To confirm ischemic changes, we performed TTC staining to monitor the existence of lesion sites in brain sections after 7 days (Figure 6d). The ischemic changes were indicated with a pale appearance while the intact sites were red. These data showed degenerative changes in sites exposed to green laser irradiation. Besides, cavity size was evaluated by stereological examination at 7, and 28 days in different groups (Figure 6e,f). Noteworthy, we did not find statistically significant differences in cavity size in all groups 7 days after therapeutic intervention (p > 0.05). On day 28, the transplantation of NSCs alone did not alter cavity size compared to the PBS‐treated stroke mice (p > 0.05). Based on our data, the application of Reelin alone or in combination with PLGA‐PEG micelles can reduce cavity size in comparison with the PBS + Stroke group (p < 0.05). Interestingly, we found that the reduction of cavity size was at the maximum levels in stroke mice that received Reelin‐loaded PLGA‐PEG micelles (p < 0.05; Figure 6e,f). These data showed that NSCs cannot alter the cavity size that occurred after the ischemic change. Simultaneous application of NSCs with PLGA‐PEG micelles or Reelin seems an appropriate strategy to diminish the extent of the lesion. The concurrent use of Reelin and PLGA‐PEG micelles heightens the therapeutic effect of NSCs.

### 3.10 Reelin‐loaded PLGA‐PEG micelles promoted NSCs differentiation in vivo

**FIGURE 7:**
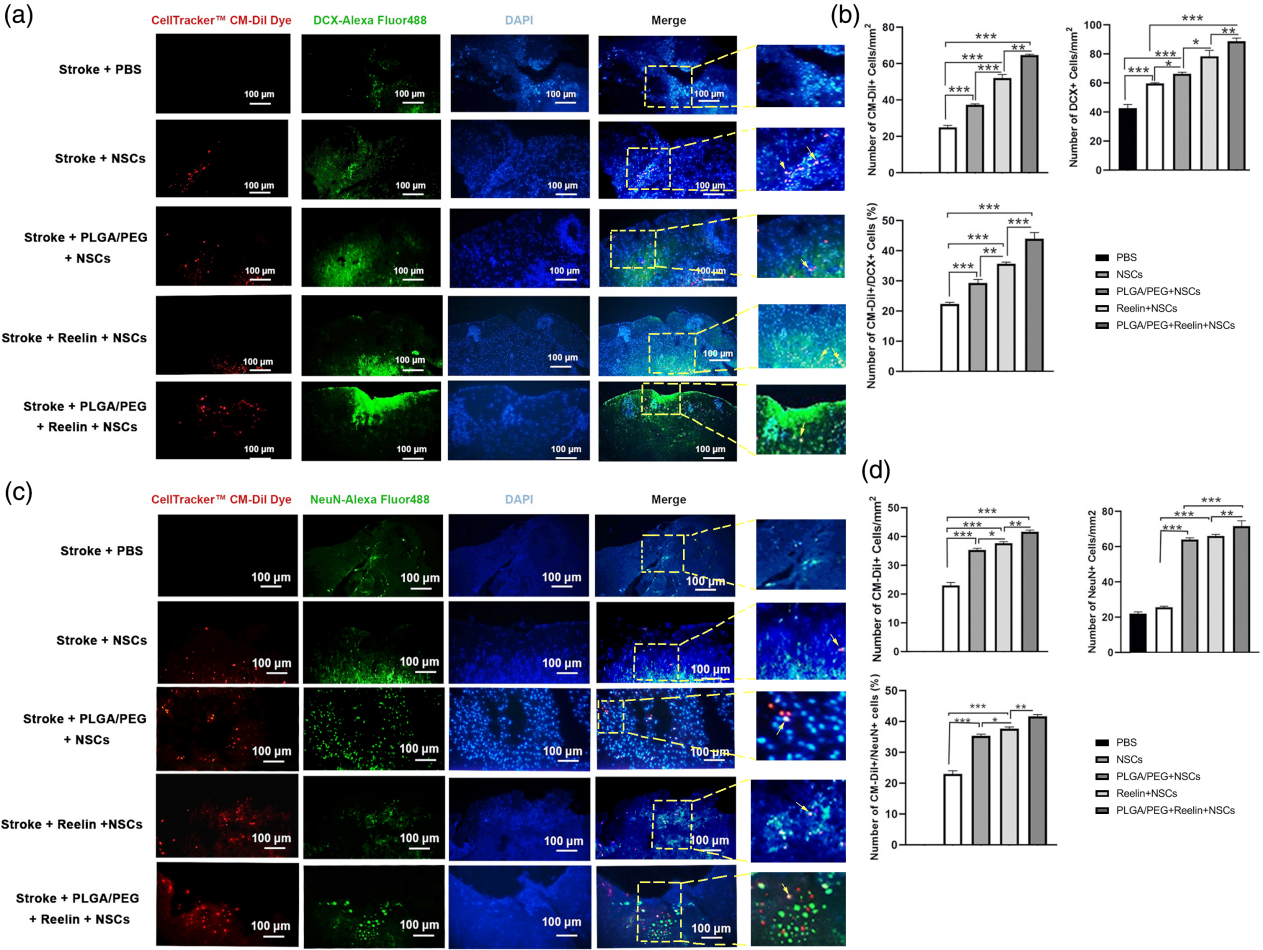
Measuring protein levels of DCX and NeuN in lesion site after transplantation of PLGA‐PEG, Reelin, and Reelin‐loaded PLGA‐PEG micelles in brain tissue (a‐d). According to data, the Reelin‐loaded PLGA‐PEG micelles can efficiently increase the percent of DCX and NeuN positive cells in the sites of injection compared to the Reelin, PLGA‐PEG, and other groups. One‐way ANOVA and Tukey post hoc analysis. *p < 0.05; **p < 0.01; and ***p < 0.001. PLGA‐PEG, polylactic‐co‐glycolic acid‐polyethylene glycol

To assess the effect of Reelin‐loaded PLGA‐PEG, micelles on NSCs migration and differentiation, we performed IF analysis (Figure 7a‐d). On Day 14, three mice were selected for the evaluation of DCX positive cells, migrating neuroblast marker, at the site of injection (Figure 7a‐b). Data showed that the number of Dil‐labeled NSCs expressed DCX was increased in all groups compared to the Stroke + PBS group. Transplantation of NSCs in PLGA‐PEG micelles loaded with Reelin increased cell neuroblast–like phenotype acquisition compared to all groups at the site of injection (*p* < 0.05). Compared to the Stroke + PLGA‐PEG + NSCs, we found significant increase in DCX^+^ cells in group was found in the Stroke + Reelin + NSCs group (*p* < 0.05; Figure 7a‐b). These data revealed that a combination of PLGA‐PEG micelles and Reelin commits transplanted NSCs into the neural lineage and increases migration neuroblasts.

Along with this analysis, we also studied the differentiation of injected NSCs toward mature neurons using NeuN staining (Figure 7c‐d). Our data showed that transplantation of Dil‐labeled NSCs using Reelin, PEGA‐PEG, and Reelin‐loaded PEGA‐PEG increased the number of NeuN^+^ cells compared to the control PBS and NSCs (*p* < 0.05). These effects were more prominent when the combination of Reelin and PLGA‐PEG was used compared to the other groups (*p* < 0.05; Figure 7c,d). These data showed that NSCs can orient toward maturity when the combination of Reelin and PLGA‐PEG was used as a supporting matrix. Taken together, the present findings imply that the cocktail of Reelin and PLGA‐PEG can promote neurogenesis by providing a suitable microenvironment and cell niche.

### 3.11 Co‐administration of NSCs with Reelin‐loaded PLGA‐PEG micelles reduced astrocytic gliosis and increased local angiogenesis

**FIGURE 8:**
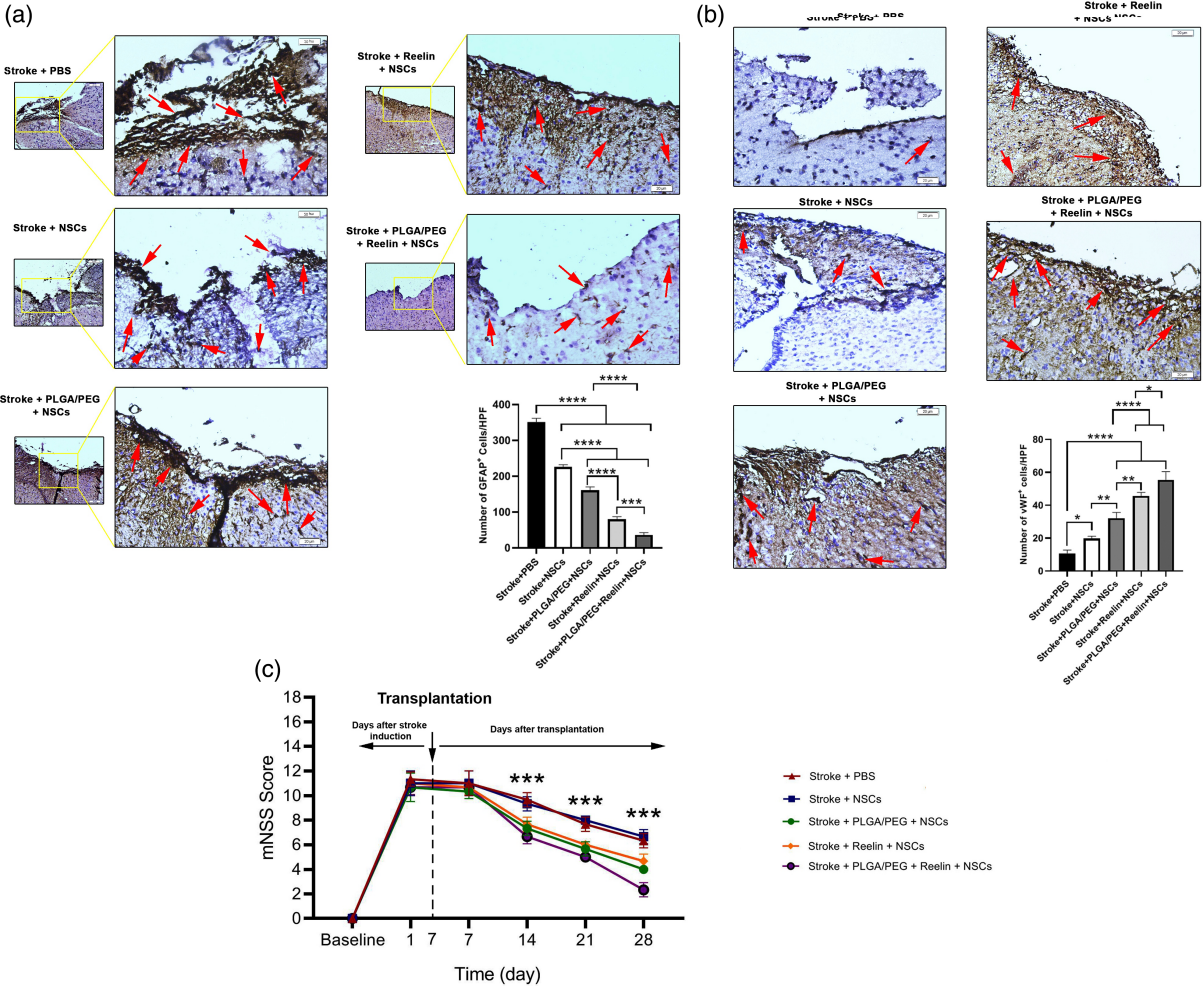
Monitoring astrocytic gliosis and angiogenesis at the site of injection using IHC analysis of GFAP and vWF factors (a,b). Data showed enhanced angiogenesis rate and inhibition of astrocytic gliosis in the ischemic region with the combination of Reelin and PLGA‐PEG micelles with NSCs (red arrows). mNSS test revealed a neurological deficit in all animals during the first 7 days after PT stroke induction (c). The injection of NSCs with PLGA‐PEG, Reelin, and Reelin‐loaded PLGA‐PEG micelles improved mNSS and reached control levels 14, 21, and 28 days after stroke induction. Data showed the superiority of Reelin‐loaded PLGA‐PEG micelles plus NSCs to alleviate mNSS. Two‐way ANOVA. ***p < 0.001. GFAP, glial fibrillary acidic protein; mNSS, modified neurological severity score NSC, neural stem cells; PLGA‐PEG, polylactic‐co‐glycolic acid‐polyethylene glycol; PT, photothrombotic

Data showed that the administration of Reelin, PLG‐PEG, and Reelin‐load PLGA‐PEG micelles with NSCs can alter the levels of astrocytic gliosis and local angiogenesis in the ischemic areas compared to the Stroke group received PBS (Figure 8a,b). According to our findings, the promotion of ischemic changes and necrosis recruited numerous astrocytes in the periphery of lesion sites. Of note, the administration of Reelin or PLGA‐PEG in combination with NSCs can significantly reduce the number of recruited astrocytes (GFAP^+^ cells) in the periphery and inside the necrotic sites compared to the control stroke group (Figure 8a,b). These features were more evident in groups after transplantation of Reelin‐load PLGA‐PEG micelles with NSCs. By contrast, we found that the reduction of astrocyte recruitment coincided with enhanced vascularization (vWF^+^ cells) when Reelin, PLG‐PEG and Reelin‐load PLGA‐PEG micelles with NSCs transplanted into the lesion sites (Figure 8c). Of note, the combination of Reelin‐load PLGA‐PEG micelles yielded significant differences in the number of vWF cells compared to groups that used Reelin and PLGA‐PEG micelles alone with NSCs (Figure 8c). These data showed that the reduction of cavity size in the ischemic brain after injection of Reelin‐loaded PLGA‐PEG micelles and NSCs occurred with induction of local angiogenesis and suppression of astrocytic gliosis into the infarct areas.

### 3.12 Reelin‐load PLGA‐PEG micelles plus NSCs improved neurological outcomes

The mean mNSS score of mice from different groups was measured 1 day after stroke and 7, 14, 21, and 28 days after hydrogel transplantation (Figure 8c). According to our data, mNSS indices were increased (between 11 and 12 score), showing the existence of functional deficits following stroke and these conditions last for 7 days. On Day 7, we transplanted micelles plus NSCs into the stroke area. As shown by the mNSS analysis, the stroke‐related neurological deficits were recovered with time in all groups (Figure 8c). We noted that the combination of NSCs with PLGA‐PEG, Reelin, and Reelin‐loaded PLGA‐PEG significantly ameliorated the mNSS scores compared to the NSCs and PBS groups 14, 21, and 28 days after the transplantation (Figure 8c). These data showed that the transplantation of Reelin‐loaded PLGA‐PEG along with NSCs supports the recovery of mNSS score 28 days after ischemic changes. No statistical changes were found between the mean mNSS scores of PBS and NSCs‐treated mice.

## 4 DISCUSSION

The delivery of autologous and allogeneic NSCs can support the restoration of injured areas in CNS in studies targeting different animal models.^47^, ^48^ Despite these advantages, most of the previously conducted clinical trials failed to achieve the therapeutic outcome in human counterpart which tempered enthusiasm in the understanding of the underlying reparative mechanisms after NSCs transplantation.^49^ On this basis, the promotion of regeneration after ischemic changes will necessitate certain tissue engineering strategies like the selection of suitably modified substrates (native ECM components or ECM‐like composites) along with NSCs to support the integration of transplanted cells, protect the neighboring host tissue, and promote revascularization into the affected areas.^50^

Here, we examined the ability of PLGA‐PEG micelles loaded with Reelin in the modulation of mouse NSCs dynamic growths, differentiation, neurite growth, and neuroregenerative potential in in vitro condition and in vivo ischemic stroke model. In this study, PLGA blocks were functionalized with the addition of PEG to increase hydrophilicity which is indicated by ^H^NMR and FTIR spectra. SEM imaging and DLS analysis revealed the synthesis of relatively homogenous spherical PLGA‐PEG micelles with appropriate zeta potential and diameter sizes. Our data showed that PLGA‐PEG micelles can harbor appropriate Reelin content indicated by Bradford analysis. The existence of electrostatic interactions between the Reelin and PLGA‐PEG micelles seems to be the main cause in the formation of Reelin‐PLGA‐PEG micelles.^51^ The previous experiments noted the fact that PLGA nanoparticles have been confirmed as the appropriate carriers for transferring the macromolecules such as genes and proteins.^52^ Meanwhile, the addition of PEG to the PLGA backbone can increase the bioavailability of nanostructure and resistance against enzymatic degradation.^52^

In vitro analysis indicated that PLGA‐PEG micelles significantly enhanced the viability of NSCs at all‐time points in comparison with the control group, and the highest effect was achieved on Day 21. This result may be partially due to the interaction of cells with the underlying substrates that stimulate surface mechanoreceptors and downstream effectors such as FAK signaling cascade.^53^, ^54^ We also investigated the viability of mouse NSCs after exposure to different concentrations of Reelin during 21 days. Noteworthy, Reelin can change the viability of NSCs in a dose‐dependent manner at all‐time points. One reason would be that Reelin can affect the progenitor cells via engaging surface very‐low‐density lipoprotein receptor (VLDLR) and downstream signaling effectors like PI3K and Cdc42.^55^

Neuronal proliferation and differentiation are essential elements for providing a neural network in vitro.^30^ We indicated that three conditions consisted of Reelin, PLGA‐PEG, and Reelin‐loaded PLGA‐PEG micelles encouraged proliferation and differential maturation of mouse NSCs after 7 and 14 days, respectively. The percentage of Ki‐67^+^ and Map2^+^ cells was at the maximum levels in Reelin‐loaded PLGA‐PEG micelles compared to the other groups. It was suggested that the activation of adaptor protein namely Disabled‐1 (Dab‐1) belonging to the Reelin signaling pathway participates in the orientation of NSCs to mature neurons and neurogenesis.^56^ The activation of Dab‐1 can trigger downstream effectors such as PI3K/Akt signaling pathway associated with cell proliferation.^57^ Furthermore, all three conditions including Reelin, PLGA‐PEG, and Reelin‐loaded PLGA‐PEG micelles provide suitable surfaces for the attachment and flattening of mouse NSCs. These changes coincided with an increased neurite number and length and branch points in Reelin‐loaded PLGA‐PEG micelles compared to the other groups. In our previous study, we noted that PLGA‐PEG nanofibers can accelerate neural differentiation and neurite growth in human neuroblastoma cells during 2 weeks in the in vitro condition.^30^ It has been shown that Reelin phosphorylates S6 kinase 1 in mTORC1/PI3K‐dependent manner, which leads to dendritic growth and branching.^58^ These data showed that substrate composed of PLGA‐PEG is eligible to support neural growth and neurite formation and the addition of ECM glycoprotein, Reelin, acts as an anchor for cell attachment, migration, and development of cellular projections.

Here, we evaluated neurogenesis and functional behavior efficiency by using a cocktail of PLGA‐PEG, Reelin, and NSCs in the photothrombotic stroke in the model of the mouse. The evaluation of the cavity size in brain sections using stereological study revealed the reparation of injured site after 28 days in mice received NSCs in combination with Reelin, PLGA‐PEG, and PLGA‐PEG plus Reelin compared to PBS and NSCs groups. Considering the stimulatory effect of Reelin‐loaded PLGA‐PEG micelles on dynamics growth and differentiation of mouse NSCs, we also monitored these effects in in vivo conditions. Similar to the in vitro data, an increase in the percent of DCX^+^ and NeuN^+^ cells was notified in transplanting Dil^+^ NSCs injected simultaneously with Reelin‐loaded PLGA‐PEG micelles. Along with the above‐mentioned effects, we also noted the increase of Dil negative endogenous DCX^+^ and NeuN^+^ cells at the periphery of the lesion site. It is suggested that Reelin can induce proliferation and migration of both exogenous and especially endogenous NSCs activity during the ischemic changes. In line with these findings, the contribution of Reelin glycoprotein in neurite outgrowth and axonal regeneration has been demonstrated previously.^59^, ^60^ Mice deficient in Reelin exhibited defective axon regeneration, indicating the implication of Reelin in axonal regeneration after nerve damage.^61^ Commensurate with in vitro and in vivo data, one could hypothesize that PLGA‐PEG micelles with Reelin promotes the commitment of NSCs toward neural lineage and adult neurogenesis. The reduction of the cavity size can be possibly related to enhanced proliferation and differentiation of transplanted NSCs into the affected area, which results in the adult neurogenesis phenomenon. Besides, it should not be neglected that the increased neurogenesis is not solely induced by proliferation, differentiation, and ECM reconstruction can also occur simultaneously. Whether the Reelin‐loaded PLGA‐PEG micelles can resist for 28 days in in vivo condition to promote adult neurogenesis or induce the restoration of injured ECM need more investigations with prolonged time. Regarding the uptake of PLGA‐PEG micelles and Reelin‐loaded PLGA‐PEG micelles, it is noteworthy that the macromolecules such as proteins or other strange substances such as nanoparticles interact with the cell membrane and are absorbed by the cells through an energy‐using process named “endocytosis”. Endocytosis is classified into two types, phagocytosis and pinocytosis. Phagocytosis occurs mainly in phagocytes like macrophages, monocytes, neutrophils and dendritic cells. Pinocytosis has two sorts of clathrin‐mediated endocytosis and the clathrin‐independent endocytosis. Clathrin‐mediated endocytosis is primarily responsible for the uptake of polymeric nanoparticles, while the internalization of micelles may be through clathrin‐independent endocytosis.^62^, ^63^

The induction of angiogenesis along with the reduction of pathological remodeling (uncontrolled astrocytic gliosis) is an appropriate strategy to restore the function of injured cells inside the ischemic sites.^64^, ^65^ Here, we found that NSCs can promote local angiogenesis and inhibit statistical correction for multiple comparisons astrocytes recruitment into the ischemic site when co‐administrated with Reelin‐loaded PLGA‐PEG micelle. Previously, the critical role of Reelin has been proved in association with neuro‐glia‐vascular regeneration.^66^ The activity of Reelin is done on the target cells via an engaging Dab‐1 signaling pathway, leading to the activation of vascular endothelial growth factor receptor 2).^60^, ^66^, ^67^ Besides, the increase of NSCs migration into the injured area occurs in the presence of Reelin possibly by the promotion of the Akt/Ras axis.^59^, ^68^, ^69^ The promotion of angiogenesis inside the micelles can provide the vascular bed that is essential for neuronal migration, cortical development, and neurogenesis.^70^ Moreover, Reelin‐mediated signaling cascades can regulate certain adhesion molecules in neuronal cells and their differentiation of NSCs to functional motor neurons.^59^ Therefore, Reelin might be an effective candidate for the restoration of brain damage and patients with hemiplegia.^59^ Based on the great body of experiments, three‐dimensional biomaterials not only offer a supportive environment for the transplanted cells, but also is capable of reducing glial scar formation.^71^ It was suggested that Reelin can stimulate the regeneration of dorsal root ganglia following axotomy.^72^ Of note, the injection of purified Reelin increases the regional density of the dendritic spine and hippocampal CA1 long‐term potentiation coincides with spatial learning and memory, showing Reelin activity on synaptic function and cognitive performance in rodents.^73^ Therefore, it can be proposed that the reduction of astrocytes in the injured sites correlates with the promotion of neurogenesis, leading to a reduction of astrocyte recruitment into the injured sites. Accordingly, mNSS scores exhibited higher functional recovery in stroke mice that received Reelin‐loaded PLGA‐PEG micelles compared to the other groups. Therefore, the orientation of transplanted NSCs toward functional neurons can alleviate the functional behavior disorders after ischemic conditions.

The present study faces some limitations that future experiments should consider. For example, the neuronal functionality of differentiated neurons in the presence of Reelin‐loaded PLGA‐PEG micelles can be analyzed using electrophysiological methods such as patch‐clamp analysis. The synaptic activity and axonal projection of transplanted NSCs with target lower neurons in the brainstem and spinal cord should be also monitored in a prolonged time. For instance, transplantation of permanently labeled cells using anterograde and retrograde tracers into the target sites allowing visualization of cell morphology, projection, and axonal development can help us to monitor time‐dependent activity and phenotype acquisition. Besides, studying the synaptogenesis between generated upper motor neurons with lower motor neurons in the different levels of brain stem and spinal cord segments can mimic the functionality and structural remodeling of CNS following the transplantation of NSCs.

## 5 CONCLUSION

This study provides a deeper understanding of the three conditions (PLGA‐PEG micelle, Reelin, Reelin‐loaded PLGA‐PEG micelles) that dictate the differentiation of neural progenitors into the mature neurons. All conditions induced cell differentiation to some extent, suggesting that the presence of PLGA‐PEG micelles with Reelin promoted efficiently NSCs proliferation, survival, and differentiation in vitro and in vivo conditions. Taken together, our study presents eligibility and suitability of Reelin‐loaded PLGA‐PEG micelles on neural tissue regeneration and functional recovery following ischemic stroke, leading to the introduction of novel approaches in the field of neural tissue engineering and regenerative medicine.

## CONFLICT OF INTEREST

The authors declare that they have no conflict oF interests.

## AUTHOR CONTRIBUTIONS

**Zahra Shabani:** Investigation (supporting); methodology (supporting); writing – original draft (equal). **Reza Rahbarghazi:** Methodology (supporting); software (lead); writing – review and editing (lead). **Tahereh Ghadiri:** Methodology (supporting). **Roya Salehi:** Methodology (supporting). **Saeed Sadigh‐ Eteghad:** Writing – original draft (supporting).

### PEER REVIEW

The peer review history for this article is available at https://publons.com/publon/10.1002/btm2.10264.

## Data Availability

Any data associated with this study are available from the authors upon reasonable request.
